# Preparation of PVA-Based Hollow Fiber Ion-Exchange Membranes and Their Performance for Donnan Dialysis

**DOI:** 10.3390/membranes9010004

**Published:** 2019-01-02

**Authors:** Mitsuru Higa, Yuriko Kakihana, Takehiro Sugimoto, Kakuya Toyota

**Affiliations:** 1 Graduate School of Sciences and Technology for Innovation, Yamaguchi University, 2-16-1 Tokiwadai, Ube, Yamaguchi 755-8611, Japan; kakihana@yamaguchi-u.ac.jp; 2 Blue Energy Center for SGE Technology (BEST), Yamaguchi University, 2-16-1 Tokiwadai, Ube, Yamaguchi 755-8611, Japan; 3 Graduate School of Science and Engineering, Yamaguchi University, 2-16-1 Tokiwadai, Ube, Yamaguchi 755-8611, Japan; i051vf@yamaguchi-u.ac.jp (T.S.); g012vf@yamaguchi-u.ac.jp (K.T.)

**Keywords:** ion exchange membranes, poly (vinyl alcohol), hollow fiber, Donnan dialysis

## Abstract

Hollow fiber type cation-exchange (C-HF) membranes and hollow fiber type anion-exchange (A-HF) membranes were prepared from poly (vinyl alcohol) (PVA)-based copolymer with cation-exchange groups and by blending PVA and polycation, respectively, by a gel fiber spinning method. In order to control the water content of the hollow fiber membranes, the membranes were cross-linked physically by annealing, and then cross-linked chemically by using glutaraldehyde (GA) solutions at various GA concentrations. The outer diameter of C-HF and A-HF membranes were ca. 1000 μm and ca. 1500 μm, respectively, and the thickness of the membranes were ca. 170 μm and 290 μm, respectively. Permeation experiments were carried out in two Donnan dialysis systems, which included mixed 0.1 M NaCl and 0.1 M CaCl_2_/C-HF /3 × 10^−4^ M CaCl_2_ and mixed 0.1 M NaCl and 0.1 M NaNO_3_/A-HF/3 × 10^−4^ M NaNO_3_ to examine ionic perm selectivity of the membranes. In the Donnan dialysis experiments using C-HF membranes, uphill transport of the divalent cations occurred, and, in the case of A-HF membranes, uphill transport of NO_3_^−^ ions occurred. C-HF and A-HF membranes had about half of the flux in the uphill transported ions and also about half of the selectivity between the uphill transport ions and driven ions in comparison with those of the commercial flat sheet cation-exchange membrane (Neosepta^®^ CMX) and anion-exchange membrane (Neosepta^®^ AMX). Yet, *IEC* of C-HF and A-HF membranes were about one fifth of CMX and less than half of AMX, respectively. Since hollow fiber membrane module will have higher packing density than a flat membrane stack, the hollow fiber type ion-exchange membranes (IEMs) prepared in this study will have a potential application to a Donnan dialysis process.

## 1. Introduction

**Figure 1:**
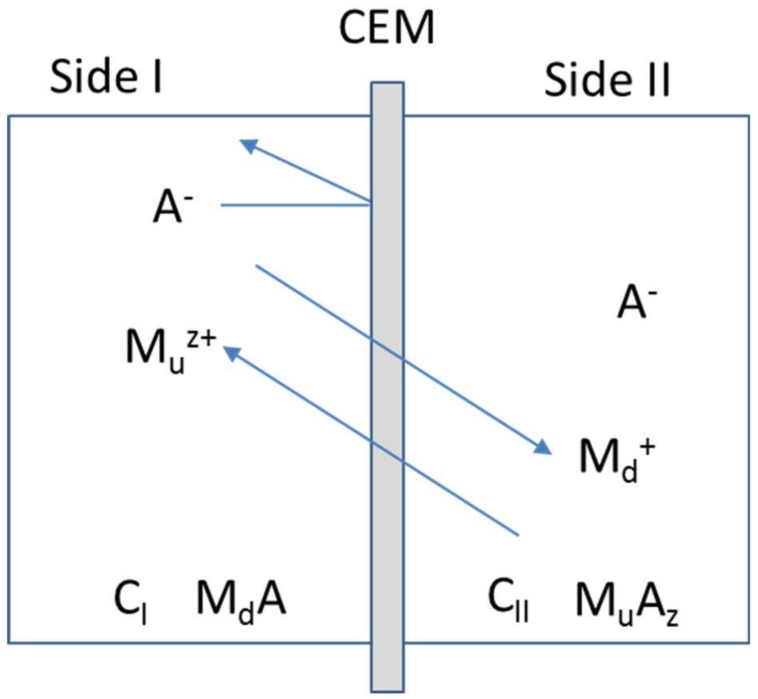
A schematic diagram of an example of Donnan dialylsis system with a cation-exchange membrane. In this case, side I and II chambers contain M_d_A as the driving electrolyte at an initial concentration of C_I_, and M_u_A_z_ at an initial concentration of C_II_ where the driving cations are the monovalnet ion, M_d_^+^, and the uphill transported ions are M_u_^z+^ ions. The system has common anions as A^-^ ions generally. In a DD system, C_I_ is higher than C_II_.

Ion-exchange membranes (IEMs) have been widly used in various industrial fields [1,2,3,4,5]. There are two separation processes using IEMs: electrodialysis (ED) driven by the applied voltage [4,5], and diffusion dialysis driven by the concentration gradient [6,7]. ED processes have been applied to table salt production from seawater in Japan [1,4,8], in the food industry such as demineralization of whey and pretreatment of fruit juice [9,10], and waste water treatment [11]. An example of diffusion dialysis processes are recovery of acids and bases used in metal treatment processes [12]. In a separation process driven by the concentration gradient using IEMs, a Donnan dialysis (DD) utilizes counter diffusion of counter-ions of IEMs through the IEM to achieve removal and concentration of other counter-ions [13,14,15]. Figure 1 shows a DD system where a cation-exhange membrane (CEM) is placed between two chambers. In the system, the electric current, *I*, must be zero. Hence, the following equation is given in the system.

(1) $$I=FS\sum^{}z_{i}J_{i}=0$$

where *F* is the Faraday’s constant. Hence, in the DD system, the following equation should be given from Equation (1).

(2) $$J_{\mathrm{Md}}+z_{u}J_{\mathrm{Mu}}-J_{A}=0$$

where $J_{\mathrm{Md}},\;J_{\mathrm{Mu}},$ and $J_{A}$ are flux of M_d_^+^ ions, M_u_^z+^ ions, and A^−^ ions, respectively.

Under an assumption that the CEM in the system has perfect permselectivity for cations, the flux of anions becomes zero. Hence, the formula below is true.

(3) $$J_{\mathrm{Md}}=-z_{u}J_{\mathrm{Mu}\;}$$

Equation (3) indicates that M_u_^z+^ ions are transported against their concentration gradient from side II to side I, which is called as uphill transport, driven by the diffusion of the driving cations known as M_d_^+^ ions. In this study, the selectivity coefficient, α, is defined from $J_{\mathrm{Md}}$ and $J_{\mathrm{Mu}}$ in terms of the following equation.

(4) $$\alpha =-\frac{z_{u}J_{\mathrm{Mu}}}{J_{\mathrm{Md}}}$$

If the system has ideal selectivity for cations, in other words, the flux of co-ions is equal to zero, α turns into unity.

DD processes can be applied for the removal, recovery, separation, and concentrations of heavy metal ions [14], and the removal of harmful anions such as nitrate ions and fluoride ions from water [15].

The DD process has been performed using a stack with a plate-and-frame configuration [1,3,7]. This configuration requires expensive flat sheet ion-exchange membranes and expensive spacers with gaskets. The process is not applied in industry mainly because of its slow kinetics [15]. The membrane module for hollow fiber type ion-exchange membranes had higher packing density (high membrane surface area in a unit volume) than the plate-and-frame stack. In addition, it does not need spacers [16]. Hence, hollow fiber membranes have been widely used for various fields such as medical use for hemodialysis as well as water treatment for purification of wastewater and seawater desalination [17,18,19,20,21]. Therefore, recently, there have been many studies on the preparation of hollow fiber type ion-exchange membranes [15,22,23,24]. Saito et al. prepared cation-exchange porous hollow fiber membranes by a radiation-induced co-grafting method [22]. Kiyono et al. prepared mixed matrix microporous hollow fiber membranes by a wet spinning technique [16].

On the other hand, recently, poly (vinyl alcohol) (PVA)-based ion-exchange membranes have been studied [25,26,27,28]. Since PVA is a semi-crystalline polymer, heat-treatment above the glass-transition temperature [29,30] increases the crystallinity of the polymer, which acts as a physical network [31]. Moreover, PVA is a polyhydroxy polymer. Hence, PVA chains can be chemically crosslinked using crosslinking agents such as glutaraldehyde (GA) [32]. Therefore, the water content of the membrane, which plays an important role in the ionic perm selectivity and mechanical strength, can be easily controlled by annealing an as-cast membrane and/or cross-linking it by using cross-linking agents.

In this study, we prepared hollow fiber type cation-exchange (C-HF) membranes and hollow fiber type anion-exchange (A-HF) membranes from a PVA-based copolymer with cation-exchange groups and by blending PVA and polycations, respectively, by the gel spinning method. Afterward, the basic property of the HF membranes was investigated. Donnan dialysis experiments were performed with the systems of mixed NaCl and CaCl_2_/C-HF/CaCl_2_ where Ca^2+^ ions were model heavy metal ions, and mixed NaCl and NaNO_3_/A-HF/M NaNO_3_ were used to examine the ionic selectivity of the prepared hollow fiber type IEMs.

## 2. Materials and Methods

### 2.1. Materials

**Figure 2:**
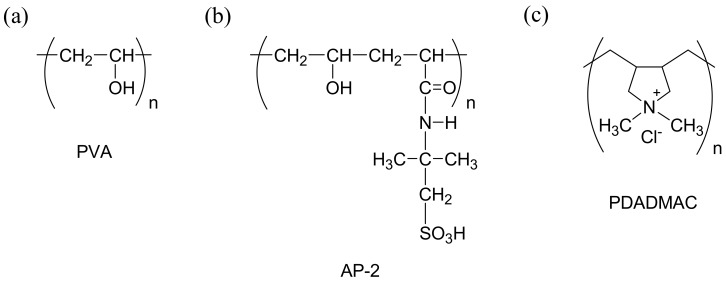
Chemical structure of (**a**) PVA, (**b**) AP-2, and (**c**) PDADMAC polymers used in this study.

Poly(vinyl alcohol) (PVA, average *M*_w_ = 85,000–124,000 g·mol^−1^, degree of hydrolysis = 99%) was purchased from Aldrich (Milwaukee, WI, USA), poly (diallyl dimethyl ammonium chloride) (PDADMAC) was purchased from Sigma-Aldrich Co. LLC. (St. Louis, MO, USA) and poly(vinyl alcohol-*co*-2-acrylamido-2-methyl propane sulfonic acid) (AP-2, average *M*_w_ = 70,000 g·mol^−1^, degree of hydrolysis = 99.5%) was obtained from KURARAY Co., Ltd. (Tokyo, Japan). AP-2 is a modified PVA that contains 2 mol% of 2-acrylamido-2-methylpropane sulfonic acid groups as a copolymer, and, hence, provides cation-exchange sites. Commercial IEMs, Neosepta^®^ CMX and AMX were obtained from the ASTOM Corporation (Tokyo, Japan). Unless otherwise specified, special solvents and reagents purchased from Sigma-Aldrich Japan Co., Ltd. (Tokyo, Japan), Nacalai Tesque, Inc. (Kyoto, Japan) and Wako Pure Chemical Industries, Ltd. (Tokyo, Japan) were used as they were. Figure 2 shows the Chemical structure of PVA, AP-2, and PDADMAC polymers used in this study.

### 2.2. Preparation of Hollow Fiber Type Ion-Exchange Membranes

#### 2.2.1. Preparation of Spinning Solution

For preparing C-HF membranes, 10 g of AP-2, 0.4 g of boric acid, 0.2 g of acetic acid, and 45.5 mL of deionized water were mixed to prepare a solution of AP-2:deionized water = 18:82 (w/w). For preparing A-HF membranes, 8 g of PVA-124, 10 g of 20 wt.% PDADMAC aqueous solution, 0.32 g of boric acid, 0.16 mL of acetic acid, and 42 mL of deionized water were mixed to prepare a solution of (PVA/PDADMAC):deionized water = 19:81 (w/w). The theoretical ion-exchange capacity calculated from the molar percent of the charged groups in AP-2, and the mixture ratio of PVA and PDADMAC were 0.42 for C-HF and 1.25 (meq/g) for A-HF membranes, respectively. These solutions were heated in a water bath at 100 °C for 5 h to obtain spinning solutions. 

#### 2.2.2. Spinning of Hollow Fiber Ion-Exchange Membranes

**Figure 3:**
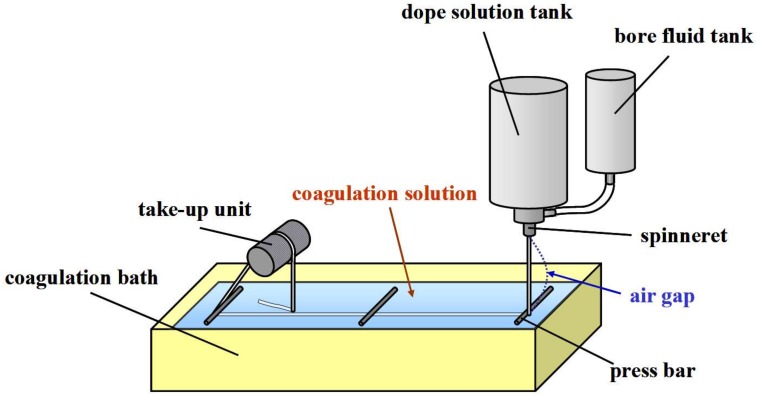
Schematic diagram of an apparatus of spinning hollow fiber type IEMs.

Spinning of hollow fiber type IEMs was performed in the same method as described elsewhere [33] by using the spinning apparatus shown in Figure 3. The AP-2 solution and the PVA/PDADMAC solution were put in the dope solution tank for preparing C-HF and A-HF membranes, respectively, and the bore fluid was put in the bore fluid tank. The spinning of the hollow fiber type IEMs was performed under the spinning conditions listed in Table 1.

After taking out the hollow fiber membranes from the coagulation bath, the membranes were immersed in a saturated aqueous sodium hydroxide solution of pH 1 in order to remove boric acid. Afterward, in order to wash boric acid and acid adhering to the hollow fiber membranes, the membranes were immersed in a saturated sodium sulfate aqueous solution. 

#### 2.2.3. Cross-Linking Processes of the Hollow Fiber Type IEMs

For physical cross-linking of the hollow fiber type IEMs (heat treatment), the prepared C-HF and A-HF membranes were immersed in ethanol to remove adhered salt. Afterward, the membranes were dried in an oven at 80 °C for 5 h under reduced pressure. In addition, heat treatment was performed at 160 °C for 10 min. The membranes after the heat treatment were immersed in a saturated sodium sulfate aqueous solution.

For chemical cross-linking of the hollow fiber type IEMs (GA crosslinking), sulfuric acid was added to a predetermined amount of saturated sodium sulfate aqueous solution so as to be pH 1. Then a predetermined amount of GA was added to prepare a GA solution. The hollow fiber type IEMs were immersed in the prepared GA solution at 25 °C for 24 h. The membranes subjected to GA crosslinking were immersed in deionized water for 1 week and were stored.

### 2.3. Preparation of a Hollow Fiber Module

**Figure 4:**
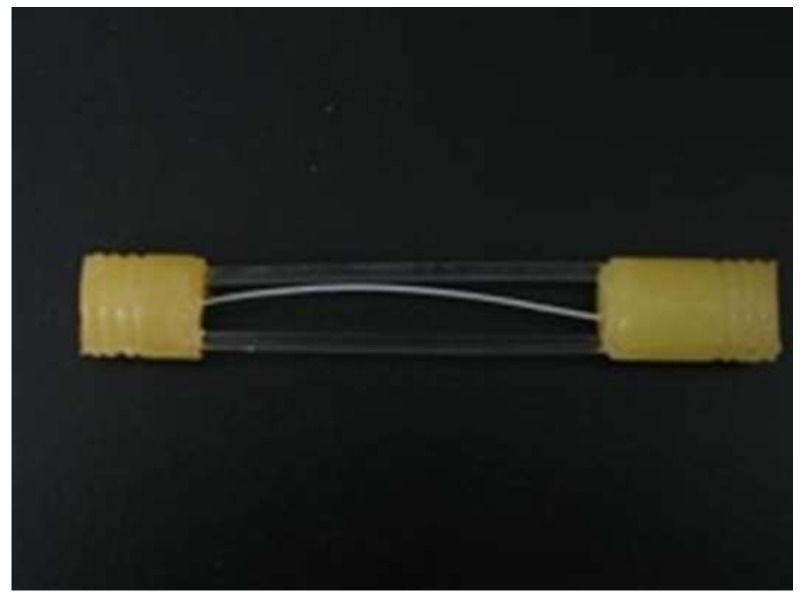
Photograph of a hollow fiber membrane module prepared in this study.

Both ends of the fabricated hollow fiber type IEMs were fixed with epoxy resin, and the end of the epoxy resin parts was cut off to obtain a flow path. Two acrylic rods were incorporated to bridge the two resin parts so as to maintain the length of the membranes as a constant. A photograph of the module prepared was shown in Figure 4.

### 2.4. Morphological Observation of Hollow Fiber Membranes

After immersing a sample membrane in deionized water, the sample was fractured in liquid nitrogen and sputtered with gold using ion sputtering equipment (HITACHI E101 ION SPUTTER, Tokyo, Japan). The sample membrane was observed with a scanning electron microscope (SEM) (KEYENCE VE-8800, Osaka, Japan) to investigate the membrane morphology. The thickness, inside diameter, and outside diameter of the membrane were measured by a SEM image.

### 2.5. Measurement of Mechanical Strength

The mechanical strength of the prepared hollow fiber type IEMs was determined by measuring stress-strain curves using a table model-testing machine (SHIMAZU, EZ-Test500N, Kyoto, Japan). In the test, specimens with a nominal 3.5 cm-gauge length were deformed by the test machine at a constant strain rate of 10 mm/min at 25 °C. The stress-strain data were determined to fail.

### 2.6. Measurement of Membrane Water Content

The water content, *H*, was measured as follows. A base membrane was weighed in the dry state after the annealing treatment, and cross-linked chemically. The cross-linked membranes were immersed in deionized water at 25 °C for 7 days. The membranes were removed from the water, dabbed with filter paper to remove excess water on the membrane surfaces, and weighed in the wet state. The volumetric water content is calculated from the weights in the wet state, *W*_w_, and in the dry state, *W_D_*, as:

(5) $$H=\frac{\left(W_{w}-W_{D}\right)/1.0}{\left(W_{w}-W_{D}\right)/1.0+\left(W_{D}/1.3\right)}$$

where 1.0 and 1.3 g/cm^3^ are the densities of water and PVA, respectively.

### 2.7. Measurement of Ion Exchange Capacity (IEC)

*IEC* of an IEM is important because the ionic transport properties such as selectivity of ions depend on the amount and species of the ion exchange groups. *IEC* is expressed as milli-equivalent per gram of membrane (meq·g^−1^ dry IEM), and was determined as follows: A sample membrane was immersed in 0.10 M KCl solution for 3 h before measuring *IEC* to exchange the counter-ions of the fixed charged groups in the IEM with K^+^ ions (C-HF membrane) or with Cl^−^ ions (A-HF membrane), respectively. Then, the samples were immersed in 0.1 mM KCl solution to remove a droplet of 0.1 M KCl solution on the membrane surfaces, and then immersed in 50 mL of 0.10 M NaNO_3_ for 12 h in order to obtain the complete exchange of K^+^ ions in the membrane for Na^+^ ions from the solution on the C-HF membrane, and to obtain the complete exchange of Cl^−^ ions in the membrane for NO_3_^−^ ions from the solution on the A-HF membrane. The concentration of K^+^ ions or NO_3_^−^ ions obtained in the solution, C*_i_*, was determined by using an ion chromatograph (Dionex ICS-1500, Sunnyvale, CA, USA). The membrane was dried under vacuum for 24 h, and was weighed in the dry state, *W_D_*. The *IEC* of the membranes was calculated using the following equation.

(6) $$IEC=\frac{C_{i}}{W_{D}}\times \frac{100}{1000}$$

### 2.8. Donnan Dialysis Experiment

**Figure 5:**
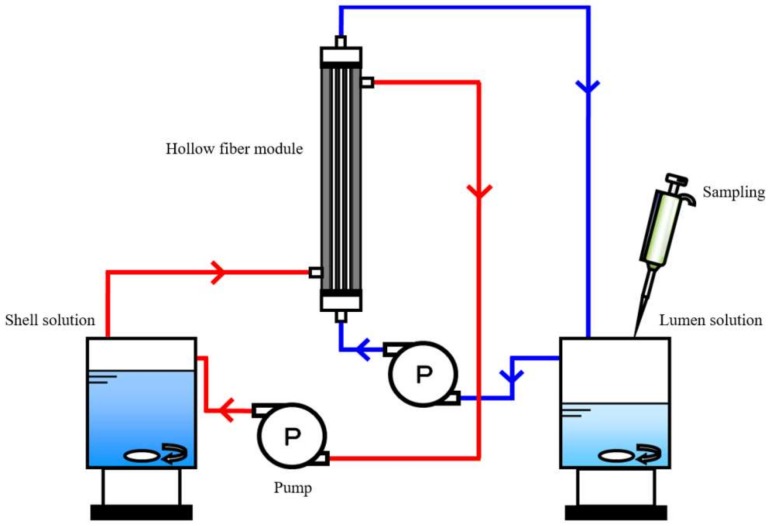
Schematic diagram of apparatus of Donnan dialysis experiments.

**Figure 6:**
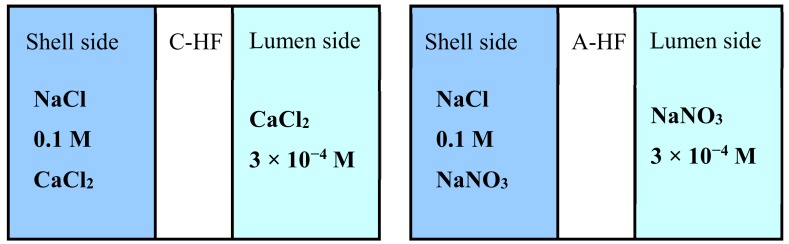
Schematic diagram of Donnan dialysis systems using the C-HF membrane and the A-HF membrane.

Ionic permeation experiments using an apparatus shown in Figure 5 were carried out using two types of the Donnan dialysis systems shown in Figure 6: mixed 0.1 M NaCl and 0.1 M CaCl_2_ (shell side)/C-HF/3 × 10^−4^ M CaCl_2_ (lumen side) and mixed 0.1 M NaCl and 0.1 M NaNO_3_ (shell side) /A-HF/3 × 10^−4^ M NaNO_3_ (lumen side). The volume of the lumen and the shell solutions were 200 cm^3^ and 800 cm^3^, respectively. The solution in the lumen side was sampled to measure the concentration of Na^+^ ions and Ca^2+^ ions with an ion chromatograph (DIONEX ICS-1500, Sunnyvale, CA, USA) in the case of the C-HF membrane, and that of NO_3_^−^ ions and Cl^−^ ions with an ion chromatograph (DIONEX ICS-2000, Sunnyvale, CA, USA) in the case of the A-HF membrane. The ionic flux of these ions, *J_i_*, was obtained from the slope of their time-concentration curves in terms of the following equation.

(7) $$J_{i}=\frac{V}{S}\times \frac{\Delta C_{i}}{\Delta t}$$

where, *V*, *S*, ∆*C*/∆*t* are the volume of the lumen side solution, the effective area of the sample membrane, and the initial slope of *i* species ions, respectively.

## 3. Results and Discussion

### 3.1. Morphology of the Hollow Fiber Type IEMs

**Figure 7:**
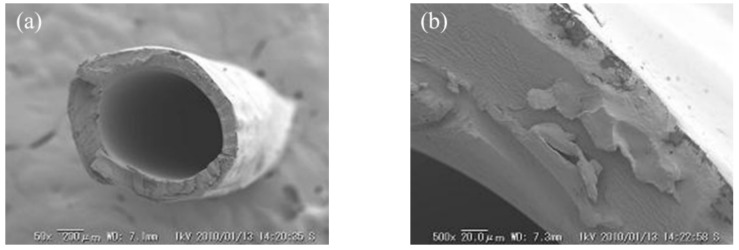
SEM cross-sectional image of C-HF membrane. (**a**) Overall membrane image and (**b**) close up image of the cross-section.

**Figure 8:**
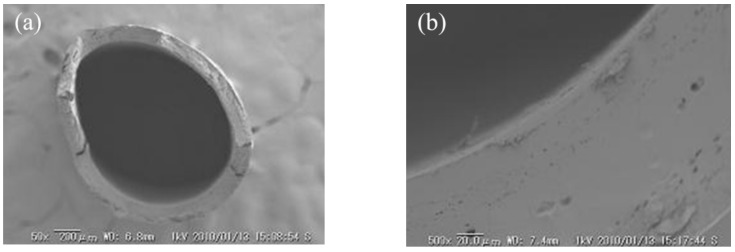
SEM cross-sectional image of the A-HF membrane. (**a**) Overall membrane image and (**b**) close up image of the cross-section.

Figure 7 and Figure 8 shows the SEM image of C-HF and A-HF membranes. A non-porous structure was shown from the cross-sectional image of SEM. The hollow fiber membranes were formed by dehydration and coagulation process, and by cross-linking with a hydrogen bond between boric acid and hydroxyl group of PVA. In addition, the physical cross-linking (annealing process) and the chemical cross-linking with GA were carried out to the membranes. Thus, the membrane matrix had a non-porous structure. The inner diameter (I.D.) and outer diameter (O.D.) and thickness of the membrane (d) determined by the cross-sectional image were listed in Table 2. The thickness of the C-HF membrane was almost equal to a commercial flat sheet CEM, Neosepta^®^ CMX, whose thickness is 160 μm and that of the A-HF membrane is about two times larger than that of a commercial flat sheet AEM, Neosepta^®^ AMX, whose thickness is 170 μm. In the annealing process of the hollow fiber type IEMs, heat-drawing was not performed. The inner diameter and outer diameter (O.D.) and thickness of the membranes will decrease when the membranes are annealed with heat drawing.

### 3.2. Water Content of the Membranes as a Function of GA Concentration

**Figure 9:**
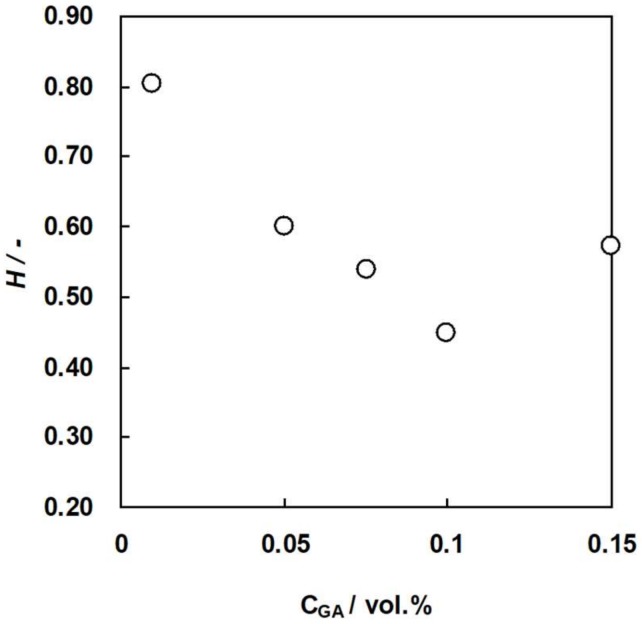
Water content, *H*, of C-HF membranes as a function of GA concentration.

**Figure 10:**
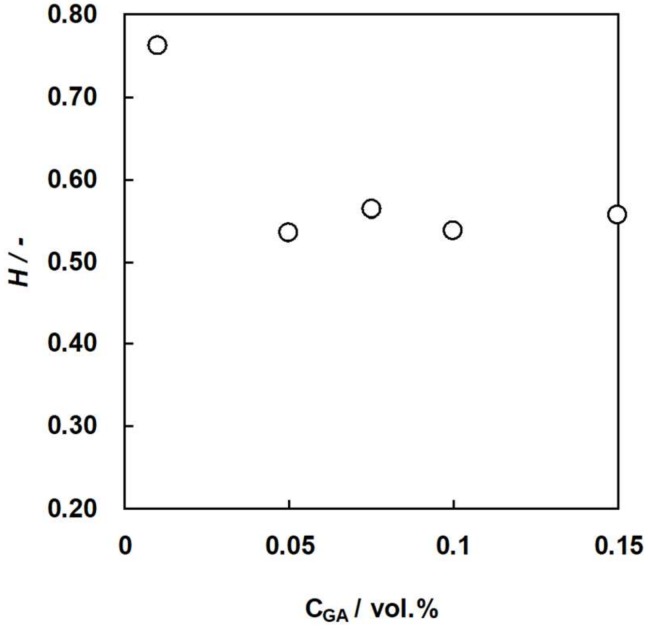
Water content, *H*, of A-HF membranes as a function of GA concentration.

Water content of an IEM is one of the several important properties because the membrane resistance, ionic selectivity, and mechanical strength of the membrane depends on the water content. Figure 9 and Figure 10 show the water content of C-HF and A-HF membranes, respectively, as a function of GA concentration. The water content of the two hollw fiber type membranes decreased with an increasing GA concentration because the water content of an IEM decreases with an increase in the number of chemical cross-linking points [34]. In general, the ionic selectivity of an IEM increases while the permeability of ions decreases as the water content decreases. In addition, the mechanical strength decreases with increasing water content. Hence, the water content of C-HF and A-HF membranes should be controlled with changing GA concentration to optimize the membrane properties.

### 3.3. Mechanical Properties of the Membranes as a Function of GA Concentration

**Figure 11:**
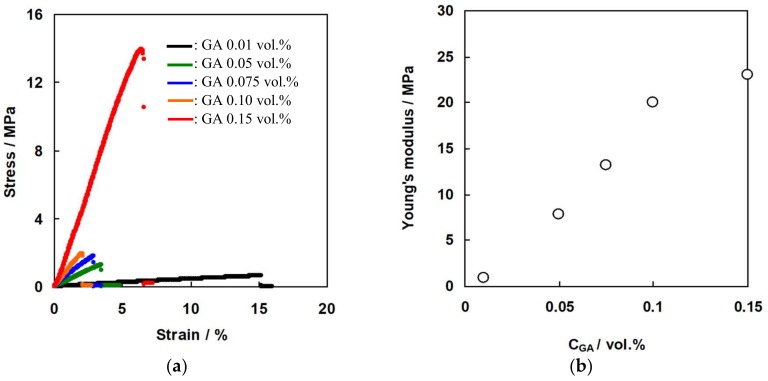
(**a**) Stress-strain curve of C-HF membranes. (**b**) Young’s modulus of C-HF membranes as a function of GA concentration.

**Figure 12:**
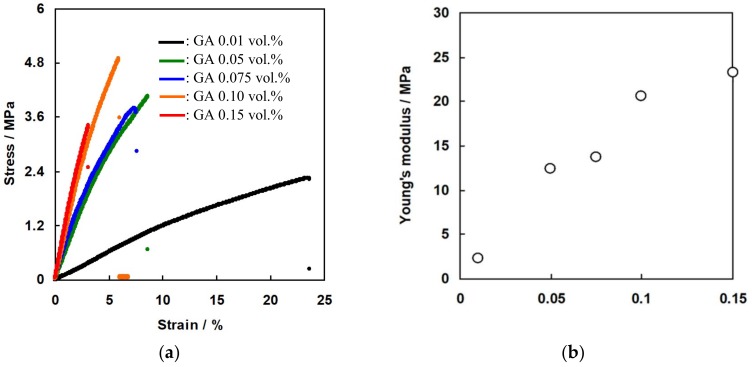
(**a**) Stress-strain curve of A-HF membranes. (**b**) Young’s modulus of A-HF membranes as a function of GA concentration.

Figure 11a shows the stress-strain curve of the C-HF membrane cross-linked with various GA concentrations. Figure 11b shows Young’s modulus of the membranes calculated from the slope of the stress-strain curve as a function of GA concentration. As expected, the maximum tensile strength at break and the Young’s modules increased with increasing GA concentration, and the membrane cross-linked with 0.15 wt% GA showed 15 MPa and 23 MPa of the tensile strength and Young’s modules, respectively. Figure 12a shows the stress-strain curve of A-HF membranes cross-linked with various GA concentrations, and Figure 12b shows Young’s modulus of the membranes. The mechanical properties of A-HF membranes have the same tendency as those of C-HF membranes, which shows that the mechanical strength increased with increasing GA concentration, and the membranes with 0.15 wt% of GA showed 3.5 MPa of the tensile strength at break and 24 MPa of Young’s modules. Both C-HF and A-HF membranes were annealed at 160 °C for physical cross-linking. M. Higa et al. reported that the degree of crystallinity of PVA-based CEMs prepared from AP-2 polymer increased with increasing annealing temperature, and the membrane annealed at 190 °C showed the higher degree of crystallinity and also had higher tensile strength and Young’s modulus than those of CEMs annealed at lower temperatures [35]. Hence, hollow fiber type IEMs annealed at more than 190 °C will have higher mechanical strength than those in the study annealed at 160 °C.

### 3.4. Ion-Exchange Capacity of the Hollow Fiber Type Ion-Exchange Membranes

Table 3 shows theoretical ion-exchange capacity (*IEC*) and measured *IEC* of C-HF and A-HF membranes as well as the measured *IEC* of CMX and AMX as a reference. The measured *IEC* of both C-HF and A-HF membranes were lower than the theoretical one. The reasons for the lower value in the measured *IEC* can be considered as follows: In the case of C-HF membranes, the molar percent is the average value, and the AP-2 polymer chains with higher molar percent than the average percent may dissolve out easier than those with lower molar percent during the spinning process because the former has higher water solubility than the latter. In the case of A-HF membranes, the linear chains of PDADMAC may dissolve out during the spinning process. *IEC* of both C-HF and A-HF membranes were lower than commercial IEMs: about one fifth of CMX, and about a half of AMX, respectively.

### 3.5. Donnan Dialysis of C-HF Membranes

**Figure 13:**
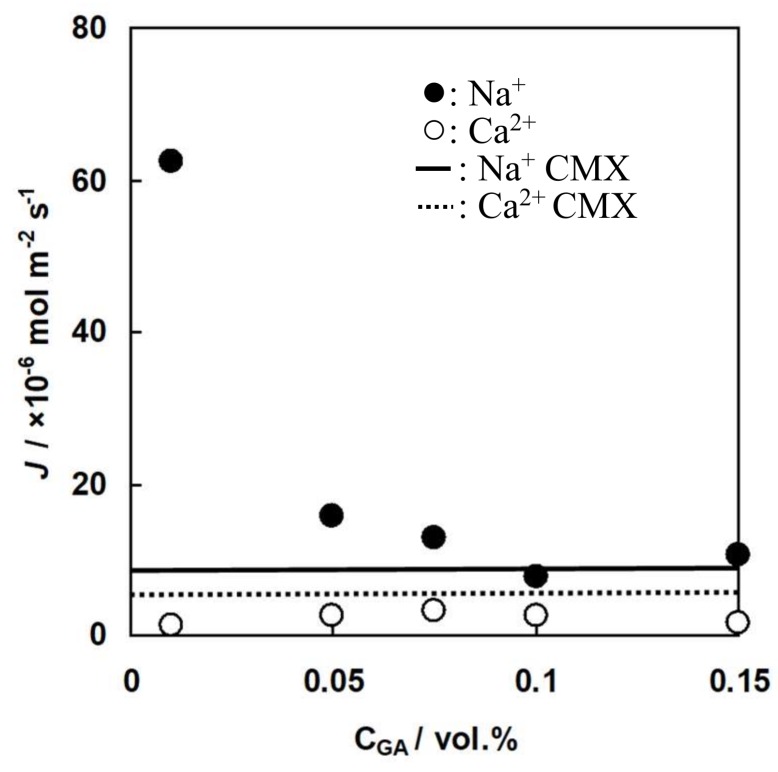
Flux of cations through C-HF membranes, *J*, as a function of GA concentration. Flux through C-HF membranes: solid circles, Na^+^ ions; open ones, Ca^2+^ ions. Flux through CMX: solid line, Na^+^ ions, dotted one, Ca^2+^ ions.

**Figure 14:**
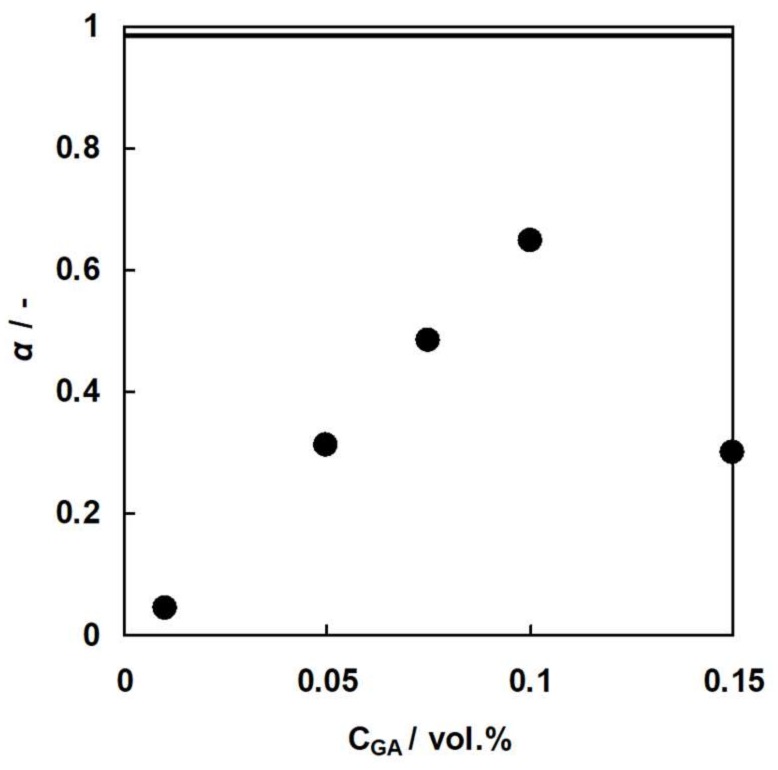
The selectivity coefficient, *α*, of C-HF membranes as a function of GA concentration. Solid circles, C-HF membranes. Solid line, CMX.

Figure 13 and Figure 14 show flux of Ca^2+^ ions and Na^+^ ions and the selectivity coefficient between the ions through C-HF membranes as a function of GA concentration where Ca^2+^ ions and Na^+^ ions are the uphill transported ions and the driving ions, respectively. Here, the flux of Na^+^ ions is defined as a positive value when the movement of the ions from the shell to the lumen sides occurs, and the flux of Ca^2+^ ions is defined as a positive value when the movement from the lumen to the shell sides occurs. Hence, Ca^2+^ ions were transported against their concentration gradient (Uphill transport), and were driven by the diffusion of Na^+^ ions. Therefore, the concentration of Ca^2+^ ions at the lumen side decreased while the concentration at the shell side increased. The flux of Na^+^ ions as the driven ions decreased showed increased GA concentration because the water content of the membranes decreased with increasing GA concentration, as shown in Figure 9. The flux of Na^+^ ions and Ca^2+^ ions through CMX under the same experimental conditions, as shown in the solid and broken lines, were 8.0 and 6.0 × 10^−6^ mol·m^−2^·s^−1^, respectively, which indicates that C-HF membranes had slightly smaller flux of Ca^2+^ ions than CMX. In the Donnan dialysis system using C-HF membranes, the following equation should be given based on Equation (1).

(8) $$J_{\mathrm{Na}}+2J_{\mathrm{Ca}}-J_{\mathrm{Cl}}=0$$

The selectivity coefficient of CMX shown as the solid line in Figure 14 was 0.98. Hence, CMX has almost perfect cation selectivity. In the case of C-HF membranes, both the flux of Ca^2+^ ions and the selectivity coefficient increased with increasing GA concentration, accepting the membrane with 0.15% GA concentration. This is due to the fact that the charge density of the membrane, which is defined as the division of *IEC* by the water content, will increase with increasing GA concentration because of the decrease in the water content of C-HF membranes. In general, the higher charge density of an ion-exchange membrane has a higher selectivity for counter-ions. Therefore, the selectivity coefficient of C-HF membranes increased with increasing GA concentration. C-HF membrane with 0.15 vol.% of GA concentration showed a lower selectivity coefficient and a higher flux of Na^+^ ions than the membrane with 0.10 vol.% of GA concentration. The reason why C-HF membrane with 0.15 vol.% had a low selectivity coefficient may be that there were some small cracks in the membrane because it had a high Young’s modulus and was brittle.

### 3.6. Donnan Dialysis of A-HF Membranes

**Figure 15:**
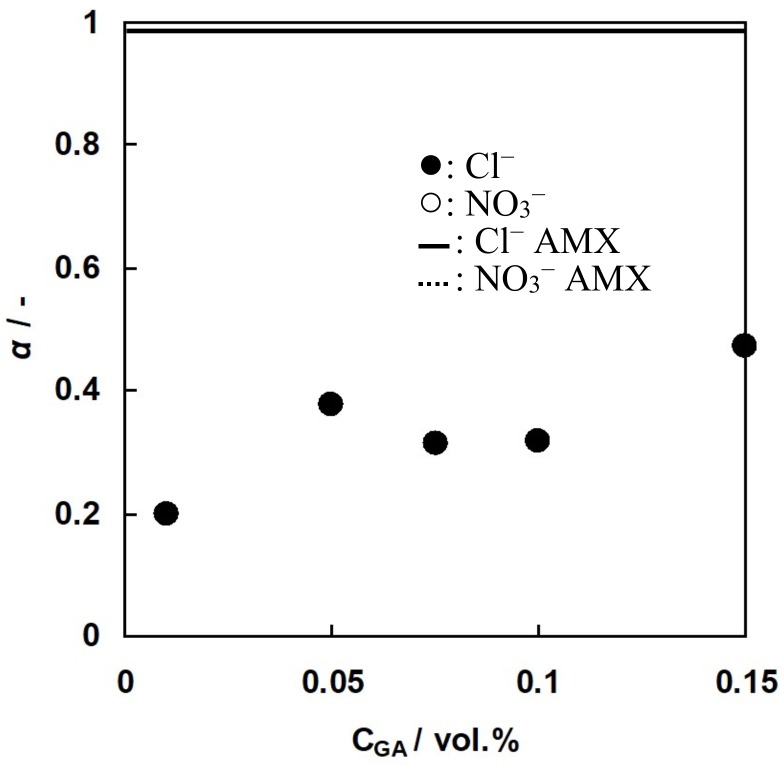
Flux of anions through A-HF membranes, *J*, of A-HF membranes as a function of GA concentration. Flux through A-HF membranes: solid circles, Cl^−^ ions. Open ones, NO_3_^−^ ions. Flux through AMX: solid line, Cl^−^ ions. Dotted one, NO_3_^−^ ions.

**Figure 16:**
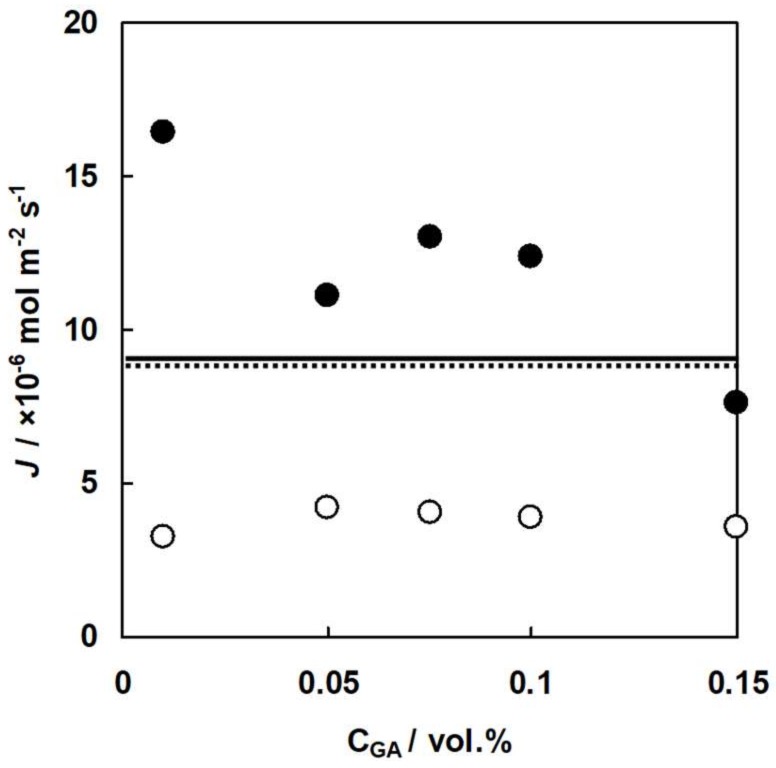
Selectivity coefficient, *α*, of A-HF membranes as a function of GA concentration. Solid circles, A-HF membranes. Solid line, AMX.

Figure 15 and Figure 16 show the flux of Cl^−^ ions and NO_3_^−^ ions, and the selectivity coefficient between the ions through A-HF membranes is a function of GA concentration where NO_3_^−^ ions and Cl^−^ ions are the uphill transported ions and the driving ions, respectively. Figure 15 indicates that uphill transport of NO_3_^−^ ions occurred, which was driven by the diffusion of Cl^−^ ions. The flux of Cl^−^ ions decreased with increasing GA concentration because the water content of the membranes decreased with increasing GA concentration, as shown in Figure 10. The flux of Cl^−^ ions and NO_3_^−^ ions through AMX shown as the solid and broken lines were 9.0 and 8.7 × 10^−6^ mol·m^−2^·s^−1^, respectively. Hence, the flux of NO_3_^−^ ions through A-HF membranes is about half of that of AMX because A-HF membranes had a larger membrane thickness (290 μm) than that of AMX (170 μm). In the Donnan dialysis system using A-HF membranes, the following equation is derived from Equation (1).

(9) $$J_{\mathrm{Na}}-J_{\mathrm{Cl}}-J_{\mathrm{NO}3}=0$$

The solid line in Figure 16 indicates that the selectivity coefficient of AMX was 0.98. Hence, AMX has almost perfect anion selectivity. In the case of A-HF membranes, the selectivity coefficient increased with increasing GA concentration. This will be due to the fact that the charge density of the membrane will increase with increasing GA concentration because of the decrease in the water content of A-HF membranes. The selectivity coefficient of A-HF membranes was less than half of that of AMX even though the selectivity coefficient of C-HF membranes was higher than half of CMX. The difference in the selectivity coefficient between C-HF and A-HF membranes can be explained as follows: distribution of divalent counter-ions inside an ion-exchange membrane is higher than that of monovalent counter-ion based on the Donnan equilibrium [36]. Hence, the selectivity coefficient of C-HF membrane for Ca^2+^ ions was higher than that of the A-HF membrane for NO_3_^-^ ions even though the former membrane has lower *IEC* than the latter one.

The hollow fiber type IEMs, C-HF and A-HF membranes, prepared in this study had about half of the flux in the uphill transported ions and also about half of the selectivity coefficient between the uphill transport ions and driven ions when compared with the commercial flat sheet IEMs. Flat sheet cation-exchange membranes prepared from AP-2 had higher performance at high annealing temperatures [35]. Moreover, Flat sheet CEMs prepared from PVA-based block copolymer, poly(vinyl alcohol-b-styrene sulfonic acid) had higher performance for CEMs than that from PVA-based random copolymer, AP-2 [37]. Hence, PVA-based hollow fiber type IEMs prepared from the block copolymer annealed at high temperatures will show higher performance than the IEMs in this study. Moreover, as mentioned above, in the annealing process of the hollow fiber type IEMs, heat-drawing was not performed. Sadao et al. reported that the degree of crystallinity in a PVA film will increase as the crystalline orientation of a PVA film increases in a heat-drawing process [38]. Hence, a PVA-based hollow fiber type IEM with a high heat-drawing ratio will have higher performance in Donnan dialysis because of its higher degree of crystallinity and thinner membrane thickness.

## 4. Conclusions

In this study, we prepared hollow fiber type cation-exchange (C-HF) membranes and anion-exchange (A-HF) membranes from PVA-based copolymer with cation-exchange groups and from a mixture of PVA and polycation, respectively, using the gel spinning method. The fundamental properties of the prepared membranes were measured. We also performed Donnan dialysis measurements to examine the perm selectivity of the membranes. 

The water content of the membranes increased with increasing cross-linker (GA) concentration at the chemical cross-linking process. *IEC* of C-HF and A-HF membranes were about one-fifth of that of CMX, and about one-half of that of AMX, respectively. 

In the Donnan dialysis experiments using C-HF membranes, uphill transport of the divalent cations occurred. Hence, the concentration at the lumen side decreased and the cations were concentrated to the shell side, which has high concentration of the driving ions. In the experiments using A-HF membranes, uphill transport of NO_3_^−^ ions occurred. The hollow fiber type IEMs, C-HF and A-HF membranes, had about half of the flux in the uphill transported ions and also about half of the selectivity between the uphill transport ions and driven ions in comparison with commercial flat sheet IEMs. 

A membrane module using hollow fiber type IEMs will have higher packing density than a plate-and-frame stack using flat sheet IEMs. In addition, PVA-based hollow fiber type IEMs with heat-drawing will have higher performance in the Donnan dialysis process than the IEMs prepared in this study. Therefore, PVA-based hollow fiber type IEMs will have a potential application in the Donnan dialysis processes.

## Figures and Tables

**Table 1:** Basic spinning conditions.

Dope solution	AP-2 and PVA/PDADMAC solution
Bore fluid	Water/Sodium hydrate/Sodium sulfate (83/2/15, w/w/w)
External coagulant	Water/Sodium hydrate/Sodium sulfate (83/2/15, w/w/w)
Spinneret dimensions (mm)	2.0/0.4 (O.D./I.D.)
Dope solution temperature (°C)	90
Bore fluid temperature (°C)	25
External coagulant temperature (°C)	25
Bore flow rate (mL/min)	32
Air gap (cm)	15

**Table 2:** The diameters and thickness of the prepared hollow fiber type IEMs.

Sample	I.D. [μm]	O.D. [μm]	*d* [μm]
C-HF	910	1080	170
A-HF	1210	1500	290

**Table 3:** Ion exchange capacities of the hollow fiber type IEMs and the commercial IEMs.

Sample	Theoretical *IEC* (meq/g)	Measured *IEC* (meq/g)
CMX	-	1.50
AMX	-	1.40
C-HF	0.42	0.28
A-HF	1.25	0.61
